# N‐Nitrosodimethylamine as an Emerging Environmental Contaminant: Sources, Analytical Advances, and Ecotoxicological and Human Health Risks

**DOI:** 10.1155/ianc/1536227

**Published:** 2026-04-07

**Authors:** Besnaci Sana, Grara Nedjoud, Khadri Sihem, Hemmami Hadia, Ali Abbas Aslam, Mohammed H. AL Mughram, Khalid J. Alzahrani, Masooma Irfan, Mahmood Ahmed

**Affiliations:** ^1^ Cellular Toxicology Laboratory, Faculty of Sciences, Badji Mokhtar University, Annaba, Algeria, univ-annaba.dz; ^2^ Laboratory of Microbiology and Molecular Biology, Faculty of Sciences, University of Badji Mokhtar, Annaba, Algeria, univ-annaba.dz; ^3^ Laboratory of Molecular and Cellular Biology, Faculty of Natural and Life Sciences, Earth and Universe Sciences, University 8 May 1945, Guelma, Algeria; ^4^ Laboratory of Interactions, Biodiversity, Ecosystems and Biotechnology Research, Department of Natural and Life Sciences, Faculty of Sciences, 20 August University of Skikda, Skikda, Algeria; ^5^ Department of Process Engineering and Petrochemical, Faculty of Technology, University of El Oued, El Oued, 39000, Algeria, univ-eloued.dz; ^6^ Department of Chemistry, Division of Science and Technology, University of Education, College Road, Lahore, Pakistan, ue.edu.pk; ^7^ Department of Pharmaceutical Chemistry, College of Pharmacy, King Khalid University, Abha, 61421, Saudi Arabia, kku.edu.sa; ^8^ Department of Clinical Laboratories Sciences, College of Applied Medical Sciences, Taif University, P.O. Box 11099, Taif, 21944, Saudi Arabia, tu.edu.sa; ^9^ Department of Mathematics, Saveetha School of Engineering (SIMATS), Thandalam, Chennai, 600124, Tamil Nadu, India, saveethaengineering.com

**Keywords:** anthropogenic activities, ecotoxicology, natural sources, NDMA, toxicity

## Abstract

Over the last few years, N‐nitrosodimethylamine (NDMA) has become a significant environmental contaminant with both anthropogenic and natural sources. The alarming profile of it can be explained by the fact that it is a genotoxic substance and easily spreads through the atmospheric and water systems. The production of NDMA takes place through a variety of pathways, such as industrial, agricultural, municipal, and atmospheric production, and its great aqueous solubility and low soil adsorption capacity enable it to pass through different environmental media easily. The review brings together modern knowledge on the origins of NDMA, its transportation in the environment via air, water, and soil, and its subsequent transformation. The analysis also compares detection methodology with some highly advanced methods of mass spectrometry that can detect trace quantities in complex environmental and pharmaceutical matrices. Ecotoxicological data point to the fact that NDMA is capable of compromising genetic integrity and reproductive fitness as well as causing systemic disruptions in aquatic and terrestrial life and has a long‐term effect on the stability of ecosystems. In humans, sustained consumption, through drinking water or ingesting contaminated food, is linked to increased cancer risk and possible negative effects on reproduction and immune health. Although progress has been achieved in treatment and control, a major unresolved challenge remains the lack of harmonized global regulatory thresholds and standardized long‐term monitoring data, which limits accurate assessment of chronic low‐dose exposure and cumulative ecological and human health risks associated with NDMA. The next steps must aim at the development of predictive molecular models, the extension of the toxicological research to neglected ecosystems, and the promotion of the international partnership in the surveillance to support the establishment of more robust regulations and protection.

## 1. Introduction

N‐Nitrosodimethylamine (NDMA) is a volatile organic compound known to be prevalent in the environment and is carcinogenic [1]. Being a member of the group of N‐nitrosamines, the group is formed by the transformation of secondary amines with nitrosating agents like nitrites. NDMA has gained more and more attention because it can be found in several environmental compartments, including air, water, soil, and biota [2]. The International Agency for Research on Cancer (IARC) classifies it as a Group 2A probable human carcinogen, underscoring the urgency of understanding its environmental behavior, toxicological effects, and pathways of human exposure [3]. The connection between NDMA exposure and various forms of human cancers (especially liver, lung, and kidney) has been strengthened by epidemiological and toxicological data [4]. Recent incidents of pharmaceutical contaminants, including valsartan and ranitidine, that occur during manufacturing or degradation with NDMA have increased regulatory and public health concerns [5, 6]. In addition to medications, NDMA is also found in food, which has led to the food safety assessment and risk analysis. For instance, a study by Eisenbrand et al. [7] demonstrated in their report the determination of nitrosamines in food by the European Food Safety Authority, highlighting that dietary exposure is a negligible route of human intake.

There are various anthropogenic sources of NDMA that are released into the environment. The NDMA is also a by‐product of industrial processes, especially in the production of pesticides, rubber, and dyes by manufacturing industries [8]. The primary contributors of NDMA contamination are wastewater treatment facilities, where NDMA is produced during disinfection by chloramination, which is a popular technique of controlling microorganisms in municipal water systems [9]. Other sources are emissions by the chemical industries, leachates by the landfills, and releases by agricultural and domestic wastewater, especially in areas with high livestock and industrial activity [10]. All these are inputs toward continuous loading of NDMA and its precursors into aquatic and terrestrial ecosystems, which have both mobility and persistence, leading to a long‐term environmental challenge.

The presence of NDMA in environmental and biological samples brings about a major challenge of analysis, considering that NDMA occurs in ultra‐trace levels, and the sample matrices are very complicated [11]. Sophisticated analysis tools have been developed to help overcome such difficulties. The methods that have significantly improved the sensitivity and selectivity of NDMA detection and made it possible to quantify it at nanogram concentrations include gas chromatography‐mass spectrometry (GC‐MS), liquid chromatography coupled tandem mass spectrometry (LC‐MS/MS), and high‐resolution mass spectrometry (HRMS) [12]. The methods are especially useful in the monitoring of NDMA in drinking water, pharmaceutical products, and wastewater effluents. Also, methods like solid‐phase extraction (SPE) have been used to prepare the samples and enhance the performance of the methods by concentrating the analytes and eliminating the interference with the matrices, particularly with aqueous and biological matrices [11]. The development and validation of such techniques have become increasingly important in response to stringent regulatory requirements following high‐profile contamination cases.

In addition to its impacts on human health, NDMA presents a serious ecotoxicological hazard [13]. It has been proven to be genotoxic and mutagenic in aquatic and terrestrial life. In water bodies, exposure to NDMA has been associated with chromosomal damage, reproductive defects, and developmental defects in fish and other invertebrates [14, 15]. Similarly, in terrestrial environments, NDMA can impact soil organisms, plants, and herbivores, particularly through exposure to contaminated irrigation water or biosolids. Invertebrate models like *Drosophila melanogaster* have also shown that NDMA has the potential to be toxic in reproductive and transgenerational toxicity [16]. The risks associated with NDMA are further exacerbated by its ability to bioaccumulate and be biomagnified through the food chain [3]. Human exposures can be through the consumption of contaminated drinking water, the consumption of fish and agricultural products irrigated with contaminated water, and possibly by inhaling the airborne NDMA in the vicinity of emission sources [17]. Since the exposure pathways are extensive, NDMA is persistent and mobile, and it is biologically reactive; therefore, it is essential to comprehensively evaluate its environmental and health risks [10]. This review aims to critically synthesize current knowledge on the sources, environmental fate, analytical detection, ecotoxicological impacts, and human health risks of NDMA, while evaluating recent advances in monitoring and regulatory frameworks to identify key knowledge gaps and priorities for risk management. In doing so, the study provides necessary information to both scientific inquiry and policy development regarding this high‐priority contaminant.

## 2. Sources of NDMA

Being a trace‐level environmental pollutant [18], NDMA distribution in different parts of the environment is due to a complex interplay of both anthropogenic and natural sources [19]. The summary of these sources is presented in Table 1, and it shows the primary natural processes and forms of human activity leading to the formation of NDMA. It is important to have a complete knowledge of these sources to be able to properly assess the risk and formulate the mitigation measures.

**Table 1:** Summary of anthropogenic and natural sources of NDMA and geographic distribution.

Source	Type	Environmental medium	Measured concentrations	Geographic location	Ref.
Wastewater treatment plant effluents	Anthropogenic	Treated wastewater	27–430 ng/L	Europe, USA, China	[20, 21]
Water disinfection (chloramination)	Anthropogenic	Drinking water	2–39 ng/L, peak: 2.9 μg/L	Ontario (Canada), California (USA)	[22, 23]
Pharmaceutical manufacturing	Anthropogenic	Industrial soils and effluents	Up to 7300 ng/L	Europe, India	[24, 25]
Pesticide and rubber production	Anthropogenic	Ambient air, contaminated soils	Qualitative presence, localized high emissions	Industrial sites, Switzerland	[26, 27]
Diesel combustion (vehicles)	Anthropogenic	Urban air	< 1–20 ng/m^3^	Urban zones, USA, Europe	[28]
Irrigation with contaminated water	Anthropogenic	Agricultural soils, groundwater	Up to 52 ng/L in groundwater	Agricultural zones (USA, China)	[29]
Microbial conversion of amines (DMA)	Natural	Nutrient‐rich soils and waters	Up to 79% of precursors detected	Forest soils, rice paddies	[30]
Atmospheric photochemical formation	Natural	Air (nighttime NOx/DMA reactions)	Local presence (not always quantified)	Polluted urban areas	[23]
Natural organic matter degradation	Natural	Eutrophic lakes, rivers	Low‐level formation observed	USA, Canada	[31]

### 2.1. Anthropogenic Sources of NDMA

Human activities represent the dominant contributors to NDMA contamination, primarily through industrial and municipal processes involving reactions between secondary amines and nitrosating agents [20]. Such conditions are particularly conducive to NDMA formation during wastewater treatment, chlorination, and the application of specific polymers in water treatment facilities [18, 32].

#### 2.1.1. Industrial and Manufacturing Sources

There are various industrial and manufacturing facilities that contribute to NDMA contamination; details of these facilities are explained below.

##### 2.1.1.1. Chemical and Pesticide Manufacturing

NDMA is known to appear as an unintended by‐product in several industrial processes, with pesticide manufacturing being one of the most prominent examples. In this sector, reactions between secondary amines and nitrites create favorable conditions for NDMA formation [33]. Health Canada’s Pest Management Regulatory Agency has reported the presence of NDMA in multiple pesticide formulations over the years. A review of more than 100 samples collected since 1990 showed that almost half (49%) contained detectable NDMA, with mean concentrations around 0.44 μg/g and maximum levels reaching 2.32 μg/g [34]. More recently, a study examining the ozonation of daminozide found NDMA production ranging from 27.6 to 248.4 μg/L at ozone doses between 0.5 and 4 mg/L. Notably, using a MIL‐100 (Fe–Mn) bimetallic metal–organic framework (MOF) catalyst reduced NDMA formation by up to 98.5%, highlighting both the reactivity of pesticide‐related compounds and the promise of advanced catalytic treatment strategies [35, 36].

Studies conducted across the world demonstrate that the problem of NDMA contamination is not confined to North America alone but is rather regarded as a worldwide regulatory and environmental challenge. In Europe, it has been reported in the wastewater effluent and treated drinking water as well. A survey carried out in Spain, Italy, and Germany revealed concentrations of between 9 and 120 ng/L in secondary effluents and concentrations of up to 35 ng/L in drinking water from conventional treatment systems. Chloramination was also found to be a major contributor to the formation of NDMA in these areas. Surface waters that receive industrial discharges in the United Kingdom have been occasionally reported to contain high levels of NDMA, up to 27 ng/L, which has led the UK Drinking Water Inspectorate to intensify its surveillance [37, 38].

In Asia, extensive monitoring programs in countries such as China and South Korea have provided further evidence of NDMA’s widespread presence. A study conducted in the Pearl River Delta reported concentrations between 2.6 and 68 ng/L in rivers influenced by municipal and industrial wastewater, with pharmaceutical and dye‐industry activities contributing significant precursor loads [12]. In the meantime, the metropolitan monitoring network in Seoul has recorded spikes of NDMA in the winter season of more than 40 ng/L, attributed to secondary atmospheric formation involving vehicular ammonia and urban NOx emissions [9]. South Asia has had its own experiences, especially when it comes to nitrosamine impurities in the pharmaceuticals. In ranitidine, 0.21 mg/kg of NDMA was found, whereas sartans were contaminated with 0.06 mg/kg of nitrosamines. Indian regulators implemented more stringent requirements, which required testing of batches of nitrosamines at levels corresponding to EMA and US‐FDA guidance [39, 40].

Although data from Africa remain limited, recent studies indicate rising concern. The results of a 2023‐2024 survey of wastewater treatment sites in South Africa reported NDMA concentrations of 11–52 ng/L in chloraminated effluents, and precursor formation was associated with the release of textile‐industry discharges [41]. The findings have prompted regulatory bodies in different parts of the world to revise their values of health‐based guidance. The RFSA [3] reaffirmed NDMA’s status as one of the most potent genotoxic carcinogens found in food. California still has one of the tightest goals of public health in the world at 3 ng/L, whereas the National Health and Medical Research Council of Australia has set an interim guideline of 100 ng/L for short‐term exposure. All these regulatory advances together highlight the increase in international awareness of the danger of NDMA and the need of international monitoring and management measures [42].

##### 2.1.1.2. Rubber, Tanning, and Dye Industries

Such industries produce NDMA through the reaction of alkylamine precursors with nitrogen oxides or nitrite salts [37]. In 98% of the samples analyzed, the airborne NDMA was detected, and the levels of the chemical were often greater than 2.5 μg/m^3^ in salt‐bath curing processes. In Germany, 0.13 μg/m^3^ was the lowest geometric mean exposure, and in Sweden, 0.34 μg/m^3^ was the highest, which clearly demonstrates the role of the secondary amine nitrosation in industrial emissions [43].

According to the Environmental Protection Agency (EPA) Technical Fact Sheet‐NDMA, in the United States, the level of NDMA in the air in industries or contaminated environments is 0.003–0.03 μg/m^3^, with occasional peak values approaching 0.1 μg/m^3^ near specific chemical manufacturing operations. The background in the nonindustrial outdoor air is normally between 0.02 and 0.19 ng/m^3^ and represents the diffuse atmospheric sources of vehicular emissions, industrial activity, and the secondary photochemical formation [44]. In China, occupational air concentrations in industrial dye and rubber manufacturing zones have been reported to range between 15 and 42 ng/m^3^, which is significantly higher than that which is usually reported in Europe or North America. The urban atmospheres under concurrent monitoring showed 0.2–1.1 ng/m^3^ of the background, which was predominantly caused by the process of secondary formation of nitrogen oxides and ammonia [9].

##### 2.1.1.3. Foundries and Fish‐Processing Facilities

Formation of NDMA in these environments occurs as a result of nitrosation of amines containing compounds in the presence of nitrites under either acidic or high temperature conditions, especially during the curing and smoking processes. These processes play a major role in the contamination of protein‐containing products, such as smoked fish, by nitrosamines [45].

##### 2.1.1.4. Municipal Wastewater Treatment Plants

Sanitization activities of municipal wastewater treatment facility, particularly chlorination and chloramination, are established routes of the formation of NDMA [22, 38]. In Ontario, a survey in 1990 found NDMA present in 27 of 39 effluent samples and as high as 0.22 mg/L. When these treated effluents are released, they enter the surface and groundwater systems, which release NDMA to the environment, bringing long‐term environmental hazards [46].

Recent investigations in various areas reveal that NDMA levels in the municipal wastewater effluents currently tend to be in the low‐to‐mid ng/L range with high occurrences mainly linked to industrial or precursor‐intensive catchments. An example is a 2024 study of five full‐scale domestic wastewater treatment plants along the Lijiang River, in China, which measured NDMA concentrations of 12–68 ng/L in secondary effluents with formation potentials of up to 240 ng/L, mostly due to the contribution of domestic wastewater and food‐processing industries [29]. Likewise, NDMA was identified in municipal effluent at 5–42 ng/L in a massive evaluation in the Pearl River Delta, which also indicates the presence of pharmaceutical residues and nitrosatable precursors [12]. European data also show better performance in terms of treatments. The NDMA concentration of 9‐120 ng/L was found in industrial and municipal effluents from Spain, and the highest levels were associated with the production of rubber and pesticides [37]. In most of the plants in Germany and the Netherlands, ozone biofiltration and advanced oxidation upgrades have further decreased the amounts of NDMA. Such contemporary data show that NDMA is still a topical micropollutant. Currently, the effluent concentrations are significantly lower than those in the survey in Ontario. The wastewater treatment technologies have significantly advanced, some industrial activities have ceased, and the chemical regulations have increased within the last 30 years.

##### 2.1.1.5. Drinking Water Treatment Plants

NDMA can also be generated during disinfection stages such as ozonation and chlorination, particularly from precursors like N,N‐dimethylsulfamide (DMS). This study demonstrated that ozonation of DMS produced NDMA with yields reaching up to 4%–40%, depending on pH and ozone dosage [47]. In addition, tertiary amines exposed to chloramination yield NDMA via mechanisms involving dichloramine and aminyl radical intermediates [48]. More recent experimental work by Li et al. [35] showed that ozonation of daminozide resulted in NDMA formation ranging from 27.6 to 248.4 μg/L at ozone doses of 0.5–4 mg/L. Complementary mechanistic studies have confirmed that ozonation of tertiary and secondary amine groups generates nitrosating intermediates, promoting NDMA formation across a range of water matrices [31]. Collectively, these findings provide strong multistudy evidence that ozonation of dimethylamine (DMA)‐containing compounds is a relevant pathway for NDMA generation in drinking water treatment and contaminated source waters.

##### 2.1.1.6. Vehicle Emissions

Emissions by automobiles, especially those with selective catalytic reduction (SCR) systems, emit ammonia (NH_3_), which is a major precursor to the formation of atmospheric NDMA [49].

##### 2.1.1.7. Pesticide Use and Agricultural Runoff

Commercial pesticides such as bromacil and 2,4‐D contain NDMA, which is readily dissolved by water and hardly adsorbed to soil, which means that NDMA can get into the environment when agricultural runoff occurs and pollute the surface and groundwater [50]. Pesticide formulations determine the mobility of such contaminants, and therefore, monitoring of the environment is necessary [51, 52].

##### 2.1.1.8. Application of Sewage Sludge

Natural NDMA precursors, biosolids (treated sewage sludge), are known to be introduced into the soil environment through land application. U.S. national survey of 80 samples of biosolids found nitrosamines (including NDMA) in 88% of the samples, and the NDMA concentrations were 504 ng/g dry weight (range: 87–920 ng/g), highlighting the common occurrence of this pollutant [53].

##### 2.1.1.9. Hospital Wastewaters

Hospital effluents have also been reported to be significant in terms of the sources of NDMA and its precursors due to the release of pharmaceuticals with DMA moieties and also because of the release of disinfectants and other medical chemicals. According to a study, NDMA levels in hospital wastewater were identified to range between 20 and 60 ng/L, and the formation capacity of up to 7300 ng/L was possible before the biological treatment process, necessitating specific remediation technologies and more stringent regulatory measures in healthcare facilities [20].

### 2.2. Natural Sources of NDMA

Although less extensively documented, natural NDMA formation is increasingly recognized through several environmental pathways.

#### 2.2.1. Atmospheric Precursor Chemistry

Recent evidence of electron paramagnetic resonance (EPR) measurements of nitrogen‐centered radicals (e.g., dimethyl nitroxide, TMH+, and UDMHr) has been found when DMA is oxidized by common oxidants such as chlorite and permanganate. These active intermediates are involved in the formation of NDMA in the atmosphere, particularly during nighttime or low‐UV conditions [18, 54].

#### 2.2.2. Microbial Production in Soils and Waters

Case–control experiments indicate that soil and aquatic microbial communities are capable of metabolizing alanine to NDMA progenitors (e.g., DMA and N‐methyl alanine), to levels of up to 79% of precursors in the experimental system. This indicates that there is a significant microbial input in NDMA production in the soil and nutrient‐enriched water bodies [55].

#### 2.2.3. Natural Organic Matter (NOM)–Mediated Reactions

The surface waters can release the NDMA as a result of chlorination or photochemical oxidation of humic compounds. Research undertaken on the Iowa River revealed that hydrophilic, high‐molecular‐weight NOM fractions provided almost 90% of the NDMA formation potential in chloramination and highlighted the importance of natural humic acids as important precursor pools [56]. All these findings hold to the belief that NDMA can be generated in situ during natural atmospheric reactions, microbial metabolism, and oxidative transformation of organic matter. As much as there is a lack of suitable quantification of environmental fluxes, such pathways have to be put into consideration when determining the background level of contamination and separating it from anthropogenic sources of contamination.

## 3. Environmental Occurrence of NDMA

The widespread occurrence of NDMA across atmospheric, aquatic, and terrestrial compartments directly reflects its dominant formation pathways. In particular, its formation via nitrosation during wastewater treatment and drinking water disinfection, along with high aqueous solubility, low soil adsorption, and susceptibility to photolytic degradation, influences its environmental fate. NDMA is a highly water‐soluble organic compound (∼1000 g/L), with a small soil adsorption coefficient (log Koc ≈ 1.0) and moderate volatility (Henry’s constant ≈ 2.6 × 10^−7^ atm·m^3^/mol), that promotes its widespread distribution between air, water, and soil compartments [46, 57]. The transformation mechanisms that regulate the environmental persistence of it are photolysis in the atmosphere (half‐life: 5–30 min) and surface waters (half‐life ∼16 m under midday solar radiation) and microbial degradation processes [58]. Although sources of anthropogenic origin, such as industrial effluents and municipal wastewater, are the main sources, natural formation mechanisms through atmospheric chemistry, microbial metabolism, or photochemical reactions play a major role.

### 3.1. Atmosphere

Industrial activities like pesticide and rubber production and emissions from diesel engines release NDMA into the atmosphere [49]. It is also capable of in situ formation by nighttime reactions between amines and nitrogen oxides, where vehicle emissions of ammonia are contributory factors to in situ formation [59]. NDMA is found in the gaseous form in the atmosphere and can be transported to tens of kilometers before deposition. Notwithstanding its potential to be widely dispersed, NDMA decomposes very rapidly when exposed to sunlight and has half‐lives in the air between 5 and 30 min [60].

### 3.2. Aquatic Environments

NDMA has a strong mobility in water bodies because it is highly soluble in water and has low affinity with the sediment particles. It gets into surface waters by direct discharge of treated or untreated effluents of the wastewater and may also develop in place when disinfection is being conducted, like chloramination and ozonation [46]. At midday, solar irradiance, NDMA photodegrades with a half‐life of around 16 min in sunlit surface waters. Nevertheless, half‐lives are reduced in turbid or organic‐rich waters and are between 33 and 86 min [58]. The migration of NDMA into groundwater can also occur, and it demonstrates a high persistence and forms a plume in both oxic and anoxic environments. Although the common concentration range of pollutants in the environment is within the range of ng/L, accidents of pollutants up to 50 ng/L have been reported. The highest concentrations in effluents caused by wastewater treatment have been up to 0.22 mg/L. It is important to note that natural photolysis can lower the levels of NDMA down to nondetectable concentrations in reuse systems by midday [23, 61, 62].

### 3.3. Soil

Volatilization plays a major role in the removal of reactive compounds in unsaturated surface soils, and field‐measured half‐lives of volatilization are 1‐2 h. Conversely, the rate of degradation in underground soils is slower, and microbial and abiotic half‐lives of degradation are between 12 and 38 days, depending on redox conditions [63, 64]. Although surface photolysis can promote NDMA degradation in surface soils, its maintenance grows with the soil depth because of the inability to penetrate light and sluggish degradation rates [65].

## 4. Geographical Limitations and Data Gaps

Although NDMA occurrence has been well documented in North America, Europe, and parts of Asia, available data from South America, Africa, and Oceania remain limited and uneven. In South America, sporadic monitoring studies, primarily from Brazil and Chile, have reported the formation of trace‐level NDMA associated with chloramination and wastewater reuse. Nonetheless, these studies are location‐based and do not have long‐term follow‐ups, such that a healthy regional risk analysis cannot be established. Equally, in Oceania, much of the available information is concentrated on regulatory reports and specific studies in Australia, which have reported NDMA as a disinfection by‐product and controlled by interim guideline values, as opposed to nationwide occurrence information. In the case of Africa, peer‐reviewed publications are not very common, and few recent reports have reported NDMA in chloraminated wastewater effluents, which indicates a large knowledge gap as opposed to a lack of risk. These regional disparities largely reflect differences in analytical capacity, monitoring infrastructure, and regulatory prioritization, rather than the true absence of contamination. As a result, systematic monitoring in South America, Africa, and Oceania must be considered as a priority in future research to allow making global assessments more balanced and providing equal opportunities to create global control regulations on NDMA [19].

## 5. Management and Public Health Implications

The physicochemical properties and environmental behavior of NDMA are not helpful in monitoring and controlling its distribution [46]. The human exposures can be based on the intake of polluted water and food, on respiratory exposure to the NDMA in the air, or on dermal contact [60]. The U.S. EPA identifies ultraviolet photolysis and advanced oxidation treatment methods as efficient methods of removing NDMA [66]. Regulatory benchmarks, such as California’s public health goal of 3 ng/L, underscore the compound’s carcinogenic potential [67]. At the federal level in the United States, the U.S. EPA has produced technical guidance on NDMA but, to date, has not promulgated a National Primary Drinking Water Regulation (MCL) specifically for NDMA. Instead, U.S. agencies and state regulators typically rely on site‐ and scenario‐specific risk assessments, health advisories, and treatment guidance (e.g., advanced oxidation and UV photolysis) to manage NDMA risk. The ATSDR toxicological profile similarly summarizes available U.S. guidance and risk characterizations. The WHO provides an authoritative background document and fact sheet describing NDMA’s toxicity and occurrence but has not set a single global numeric drinking‐water guideline for NDMA; WHO emphasizes the carcinogenicity of NDMA and the need to balance disinfection benefits with by‐product control while encouraging national adoption of monitoring programs informed by local risk. Many low‐ and middle‐income countries therefore reference WHO guidance (or national risk assessments based on the WHO framework) when formal national values are absent [68]. Effective management strategies should prioritize the reduction of precursor emissions and the implementation of robust treatment technologies across municipal and industrial wastewater systems.

## 6. Analytical Techniques for the Detection of NDMA: A Comprehensive Survey

NDMA needs very sensitive and specific analytical methods that can reach down to the ng/L limits of detection [40]. The possibility of NDMA being formed as an intermediate during the sample preparation also makes analytical detection more complex [69]. Regulatory attention (heightened by contamination events such as those involving ranitidine) has spurred the development of robust, matrix‐specific analytical methodologies. A combination of advanced chromatography and MS is reliably used to detect NDMA. This approach delivers the precise sensitivity, specificity, and regulatory compliance required for accurate results [70, 71]. The following sections provide a detailed overview of the most widely adopted analytical strategies.

### 6.1. GC‐MS

The GC‐MS is an established technique for identifying volatile compounds like NDMA [72]. The method allows the separation of the analytes according to their volatility and reactivity with the chromatographic stationary phase and then their identification and determination by detection using a mass spectrometer [73]. A secondary stage of the mass analysis, the tandem configuration (GC‐MS/MS), increases selectivity and removes matrix interferences [74]. GC‐MS/MS is especially applicable to environmental matrices and pharmaceutical products, where NDMA is volatilized, giving it an analytical edge [74, 75]. Several studies pointed to the incorporation of NDMA in regulatory measures to detect nitrosamine in the active pharmaceutical ingredients (sartans, ranitidine, and metformin) [40]. One way of combining isotope, dilution, strict sample preparation, protein precipitation, and cleanup with the use of activated charcoal cartridges has been devised [76]. The method revealed the limits of detection ranging from 0.07 to 0.3 μg/kg and limits of quantification from 0.3 to 0.9 μg/kg. The accuracy and precision reported were 95.0–105 and 0.4–2.7, respectively, which demonstrates the strength of the method. For instance, concentrations of 4 μg/kg in valsartan and 12 μg/kg in ranitidine were quantified. Methodological refinements have targeted analytical artifacts, notably NDMA formation during thermal degradation, which played a critical role in the ranitidine recall. Maximization of the injection parameters and oven temperatures has been useful in order to avoid false positives [77].

### 6.2. LC‐MS/MS

Nonvolatile or thermally sensitive compounds can be analyzed by LC‐MS/MS, and they are not degraded. This is especially significant in NDMA analysis in some pharmaceutical matrices, e.g., ranitidine. In this method, analytes are separated by LC and detected by a series of reactions in a tandem mass spectrometric apparatus, usually in the positive ion electrospray department [40]. The excellent aqueous solubility of NDMA ensures that LC‐MS/MS has a high likelihood of application with complex water systems, such as drinking water, wastewater, and surface or groundwater [32]. A strong LC‐MS/MS technique was established, and SPE was used to determine the amount of NDMA in different samples of water. Their protocol had a quantification limit of 2 ng/L, which was determined on a Thermo Scientific Quantum Access mass spectrometer with an Accela Ultra Pressure LC and a Hypersil Gold column. Both quantification and confirmation were done using MRM transitions, making the analysis reliable. Notably, the technique is not based on HRMS, and hence the operation cost and complexity are lower, even though the method maintains high accuracy with different sample types [70]. More recently, LC‐MS/MS has been extended to biological matrices, quantifying the NDMA in human plasma and urine, demonstrating excellent method performance in complex biological systems [78]. The LC‐MS/MS can be used as a primary instrument of analysis because of its ultra‐low level of detection, which is usually about the low ng/L range, and thus regulatory compliance and environmental surveillance [79]. LC‐HRMS is finding popularity in the quality control and regulation of pharmaceuticals. The US‐FDA has developed an LC‐HRMS method demonstrated to detect NDMA in ranitidine, which is based on the ICH Q2(R1) principles of validation. The procedure showed a limit of detection of 0.32 ng/mL, a limit of quantification of 1.0 ng/mL, and a quantification range of 1.0 up to 100 ng/mL [80]. In contrast to GC‐MS, LC‐HRMS has no thermal degradation artifacts and is therefore particularly appropriate for thermolabile compounds such as ranitidine. Moreover, its capability to scan completely and retrospective data analysis, which allows complete impurity profiling, increases its use in targeted and untargeted analysis [77].

### 6.3. SPE Coupled With Chromatography

Despite the high sensitivity of GC‐MS and LC‐MS/MS methods for NDMA determination, analytical accuracy is often challenged by sample preparation and matrix effects. NDMA occurs at ultra‐trace levels, and coextracted organic matter, residual disinfectants, and excipients can cause ion suppression or enhancement, especially under electrospray ionization conditions. SPE is a very important sample preparation method that is employed extensively in detecting NDMA, especially in the environmental and pharmaceutical matrices, where very small quantities of the sample have to be determined. The low environmental concentrations of NDMA (usually in the range of ng/L), which are facilitated by the high solubility of NDMA in water, and the utilization of SPE allow proper pretreatment of samples with preconcentration and matrix cleanup before subjecting the sample to chromatography [81]. The reversed‐phase sorbents, e.g., C18 or hydrophilic–lipophilic balanced cartridge, are typically utilized to capture NDMA in large volumes of water or aqueous samples and then eluted in organic solvents, e.g., methanol [82]. Optimized SPE protocols have shown recovery of above 90% and a low limit of quantification (2 ng/L with LC‐MS/MS), reduced matrix interferences, and better reproducibility. However, strict control of operational parameters such as sample pH, flow rate, and drying steps is essential to prevent analyte loss or artifactual NDMA formation during extraction [70].

In a different study, an Inertsil ODS‐3 column using a methanol–water gradient at 228 nm was used in the HPLC‐DAD method, which is meant to be used to perform a rapid, sensitive, and specific analysis. It exhibited good linearity (*R* ^2^ = 0.994) at 0.0091–0.0295 mg/kg, high precision (1.2% CV), and accuracy (94.5%–103.1% recovery). Its detection and quantitation limits (LOD: 0.0027 mg/kg and LOQ: 0.0091 mg/kg) were significantly below the FDA acceptable level of 0.3 mg/kg, indicating high sensitivity. The use of the technique showed that valsartan samples of Zhejiang Huahai (2012–2017) contained high levels of NDMA (mean 59.3 mg/kg), which were above the limits, but newer samples (after November 2018) and those of other suppliers contained insignificant or no contamination. The LC‐MS/MS technique was used as a confirmatory assay with the SCIEX Triple Quad 6500+ system using a Phenomenex Luna column (C18) and a formic acid–water/methanol gradient. This technique also had high linearity (*R* ^2^ = 0.9948), precision (CV 2.4%), and accuracy (109%, average recovery). The sensitivity of it was further supported by its LOD (0.0021 mg/kg) and LOQ (0.0070 mg/kg). Results of the LC‐MS/MS always verified the HPLC‐DAD method, especially of the newer, less contaminated batches of valsartan. Both of those approaches are considered efficient, and HPLC‐DAD is more appropriate for regular quality control, and LC‐MS/MS is more effective to confirm the success of further manufacturing changes and to point out the problems with contamination in the past [83].

A comparative evaluation of analytical techniques reveals that LC‐MS/MS provides superior sensitivity and reliability for the routine monitoring of NDMA, with limits of detection typically below 2 ng/L. In contrast, GC‐MS/MS remains advantageous for volatile matrices and confirmatory analysis. LC‐HRMS provides additional selectivity and retrospective screening capability but is associated with higher operational complexity and cost. Consequently, method selection should be matrix‐specific and guided by the regulatory purpose, required detection limits, and the availability of quality‐control infrastructure. Overall, harmonization of sample preparation protocols, systematic assessment of matrix effects, and intermethod comparison studies are essential to ensure data comparability across laboratories and surveillance programs, particularly in the context of long‐term environmental monitoring and pharmaceutical quality control.

## 7. In Silico Studies of NDMA to Evaluate Biological Reactivity

Computational methods, such as in silico modeling and molecular docking, are useful complementary methods of assessing the environmental behavior and biological reactivity of NDMA. The means allow predicting the genotoxic capability, especially by modeling the interactions of NDMA with minor grooves in DNA, which can help explain the mechanism of mutagenicity [84, 85].

Two other molecular docking studies to examine NDMA adsorption to engineered surfaces include MOFs and functionalized graphene, which can be used to develop selective adsorbents and sensing platforms [14, 86]. Moreover, the methods facilitate the modeling of NDMA formation by precursors of chloramination under conditions of varying water treatment [33]. Recent studies that use a hybrid density functional theory (DFT), docking, and molecular dynamics (MD) simulations have confirmed that NDMA–DNA complexes in the presence of hydrogen bonding are stable, which is an added coverage of its genotoxic profile [85]. Docking programs, like AutoDock Vina and PyRx, originally built for pharmaceutical discovery, are being used to study environmental contaminants and will aid the development of efficient remediation systems [86]. In addition, quantitative structure–activity relationship (QSAR) models and ADMET predictions, which are extensively applied in the pharmacokinetics field, can be modified to determine the bioaccumulation potential, persistence, and organ toxicity of NDMA. These calculating systems present a holistic, mechanistic outlook on the risk of NDMA on the environment and health.

## 8. Ecotoxicological, Genotoxicological, and Health Effects of NDMA

NDMA is an emerging environmental pollutant that is of major concern because of its prevalence, high aqueous solubility, and strong genotoxic potential. Besides being a known carcinogen in humans, NDMA has serious risks to nontarget organisms in aquatic and terrestrial systems. Not only its environmental persistence and mobility cause these risks, but also the ability to engage with biological macromolecules and cause DNA damage, cell dysfunction, and reproductive impairment with possible transgenerational effects [64].

NDMA has its toxicity by acting on cytotoxic metabolism through the cytochrome P450 enzymes (especially the CYP2E1) to metabolize into the highly toxic methyl diazonium ion, a DNA‐alkylating species. This metabolite causes O^6^‐methylguanine, N^7^‐methylguanine, and other methylated adenine adducts, which cause mutagenesis via mispairing. In addition to DNA modification, NDMA and its metabolites may also react with proteins, lipids, and other macromolecules via alkylation and oxidative reactions and cause cellular stress, dysfunctional protein activity, and hepatotoxicity [87–89]. Taken together, these processes support the known NDMA genotoxic and carcinogenic effects.

In addition to that, NDMA rarely operates singly in the environment. It is often found in combination with other anthropogenic pollutants, and synergistic effects are found with chloroacetic acid in aquatic plants, enhancing oxidative stress and ecological harm [84]. These interactions highlight the need to have integrative risk assessments in which the role of NDMA is taken into account in the complex mixtures of contaminants.

### 8.1. Effects of NDMA on Aquatic Organisms

NDMA has severe ecotoxicological hazards to water bodies because it is persistent, soluble, and can be bioaccumulative. However, even though NDMA does not partition to any significant extent into the sediment, its stability in the water body makes it easier to expose aquatic organisms to chronic exposure, especially in areas where wastewater and disinfection by‐products are released. The foregoing investigation of groundwater has recorded NDMA levels of up to 52 ng/L in the agricultural lands, which represent the risks in long‐term exposure of aquatic life [21]. In addition to the overall toxicity, NDMA has significant genotoxic and developmental effects. The NDMA leads to genetic mutations in fish and invertebrates, which interfere with the genomic integrity after exposure to NDMA in aquatic environments. The mutations disrupt reproductive fitness and cause developmental defects, such as larval and adult deformities. Genetic diversity is also under severe threat by NDMA because it leads to chromosomal aberrations as well as a lack of intrapopulation variability. Such loss of genetic diversity weakens the ability of species to withstand environmental stress and reduces adaptive potential to these stresses, which could destabilize whole aquatic ecosystems in the long run [12]. To make these effects worse, when NDMA coexists with other contaminants like chloroacetic acid, the compound is shown to have synergistic toxicity, which enhances the degree of ecological destruction, especially in submerged macrophytes. As an example, the photosynthesis impairment, increased oxidative stress indicators (SOD, POD, and GSH), and a disturbed cellular ultrastructure were observed in *Vallisneria natans* upon exposure to NDMA (0.1–10 mg/L) and chloroacetic acid [90]. These results of individuals are in line with larger findings that are summarized in Table 2, and this table summarizes the ecotoxicological impact of NDMA on diverse aquatic species. The occurrence of NDMA in situ during disinfection of water highlights the continuing problem of managing its occurrence in the water bodies [32]. These impacts are a pointer to the fact that there is an urgent need to constantly monitor and implement management measures to protect biodiversity in aquatic environments.

**Table 2:** Summary of ecotoxicological effects of NDMA on aquatic and terrestrial organisms.

Organism/group	Exposure conditions	Observed effects	Effect concentrations	Ref.
Algae (freshwater species)	Chronic exposure in lab conditions	Growth inhibition, reduced photosynthetic activity	LOEC: 0.025 mg/L	[91]
*Daphnia magna* (water flea)	Acute exposure (48–96 h)	Mortality, reduced reproduction	LC50: 7.76 mg/L (NDPA), 300 mg/L (NDMA)	[91]
*Gammarus limnaeus* (amphipod)	Acute exposure (96 h)	Mortality	LC50: 300 mg/L (NDMA)	[91]
*Austropotamobius pallipes* (crayfish)	Acute exposure (96 h)	Mortality	LC50: 2250 mg/L (NDMA)	[91]
Rainbow trout (*Oncorhynchus mykiss*)	Chronic dietary exposure (52 weeks)	Hepatocellular carcinomas	Lowest observed effect: 200 mg/kg diet	[91]
Fathead minnow (*Pimephales promelas*)	Chronic exposure	Growth effects	IC_50_: 0.2–2.0 mg/L (CL‐20)	[91]
Zebrafish (*Danio rerio*)	Chronic exposure (4 weeks)	Reduced body weight, increased mortality	LOEC: 1 mg/L (growth), 9.6 mg/L (mortality)	[91]
*Procambarus clarkii* (crayfish)	Chronic exposure (6 months)	Degeneration of the antennal gland, hyperplasia of the hepatopancreas tubules	100–200 mg/L	[91]
Fish (general)	Various exposures	Immunological responses, oxidative stress, and developmental toxicity	Variable, often low μg/L to mg/L range	[23, 60]
Aquatic plants (*Vallisneria natans*)	Exposure to NDMA^+^ chloroacetic acid	Impaired photosynthesis, oxidative stress, and ultrastructural damage	0.1–10 μg/L	[90]
*Drosophila melanogaster* (terrestrial insect)	Chronic exposure	Transgenerational reproductive and developmental toxicity	Not quantified	[16]

### 8.2. Effects of NDMA on Terrestrial Organisms

The ecotoxicological effect of NDMA in the terrestrial systems has been poorly defined because of a lack of direct toxicological information. Nevertheless, some of the studies provide useful information on its future in the environment and its possible hazards. As it has been noted, the half‐life of NDMA in soils differs depending on the vegetation and soil composition and takes 4.1 days in turfgrass‐covered soils and 22.5 days in the tree canopy. The degradation rate of NDMA, the importance of microbial activity, and organic matter are also factors [64]. Volatilization accounted for over 75% of NDMA loss within four hours postapplication, while leaching was minimal, suggesting a primary loss through atmospheric redistribution under specific soil moisture conditions. A study confirmed microbial degradation of NDMA following first‐order kinetics, with greater degradation efficiency at lower concentrations. Biodegradation rates were not significantly enhanced by nutrient supplementation and remained consistent across diverse microbial communities [63]. Although NDMA concentrations up to 10 mg/L did not elicit acute microbial inhibition, concerns persist over chronic sublethal effects, particularly in soils repeatedly exposed to biosolids or contaminated irrigation water. Potential impacts include disruption of microbial community structure, impaired nutrient cycling, and bioaccumulation through terrestrial food webs. Groundwater studies in agricultural settings have reported NDMA levels up to 52 ng/L [21], reinforcing concerns over environmental exposure and its implications for soil biota. Emerging evidence suggests that NDMA’s genotoxicity extends to terrestrial organisms [16]. Though extensively studied in aquatic systems, such genotoxic effects, including chromosomal abnormalities and loss of genetic diversity, are likely to affect terrestrial fauna under chronic low‐dose exposure. The co‐occurrence of NDMA with other toxicants intensifies these effects, as observed in macrophytes exposed to NDMA and chloroacetic acid [90]. Even after degradation, residual biological activity may persist in soils, posing enduring ecological risks [64]. These ecotoxicological observations, along with others affecting terrestrial organisms, are presented in Table 2. These findings underscore the urgent need for targeted ecotoxicological studies in terrestrial environments, along with rigorous monitoring and regulatory oversight [12, 41].

Although most NDMA genotoxicity studies focus on aquatic organisms, limited data are available for terrestrial organisms. For example, chronic NDMA exposure in *Drosophila melanogaster* has been shown to reduce fecundity and induce transgenerational developmental toxicity, indicating that NDMA can affect terrestrial invertebrates under controlled conditions (0.1–1 mM NDMA). In plants, *Allium cepa* root assay studies have demonstrated chromosomal aberrations in meristematic cells at 10–50 mg/L NDMA or nitrosamine exposure, supporting potential genotoxic impacts on terrestrial vegetation [84]. NDMA has also been detected in biosolids applied to soil (1.4–27 μg/kg dry weight), providing a realistic exposure pathway for soil organisms [53, 92]. These terrestrial observations remain limited; therefore, links between aquatic and terrestrial effects should be considered inferential and warrant a targeted study.

### 8.3. Impacts of NDMA Bioaccumulation on Human Health

NDMA bioaccumulation in fish, produce, and animal products presents a major pathway for human exposure and associated health risks. In addition to its proven carcinogenicity, chronic exposure to NDMA has been associated with reproductive toxicity, immunosuppression, and genetic mutations, which underscores the importance of systematic surveillance and regulatory vigilance [32]. Consumption of contaminated water and food is a major exposure route with severe consequences on the health of the population [16]. NDMA causes damage to DNA, which results in mutagenesis and cell abnormalities that predispose one to cancers, especially liver, lung, and kidney cancer [12].

#### 8.3.1. NDMA Bioaccumulation in the Food Chain and Associated Human Exposure

NDMA bioaccumulates across trophic levels via contaminated water, soils, and biota. The aquatic organisms take in NDMA directly through the water, enabling it to be transferred to the upper trophic levels by ingestion. At the same time, crops irrigated with contaminated water or planted on contaminated soils with NDMA have the ability to accumulate NDMA, thus exposing herbivorous and human beings to the contaminated water in the diet. A high risk of chronic exposure, in turn, is a major risk of such bioaccumulation that maximizes the concern of the potential carcinogenic, hepatotoxic, and systemic effects [93, 94]. Elevated concentrations of NDMA have been reported in drinking water, fish, and farm products, and these confirm the urgency of increased regulatory measures and monitoring [32, 64, 90]. Table 3 provides representative results in terms of main species in aquatic and terrestrial food chains that can demonstrate the bioaccumulation capability of NDMA and the risk of exposure by means of trophic transfer.

**Table 3:** Bioaccumulation of NDMA in aquatic and terrestrial food webs: key species and observed values.

Species/group	Trophic level	Environmental medium	Bioaccumulation metric	Observed values	Geographic location	Ref.
Phytoplankton	Primary producer	Freshwater	Bioconcentration factor	10–100 L/kg (Wet weight)	Various freshwater systems	[95]
Zooplankton	Primary consumer	Freshwater	Bioaccumulation factor	50–200 L/kg	Various freshwater systems	[95]
Small fish species	Secondary consumer	Freshwater	Biomagnification factor	2–5 (relative to prey)	Lake Ontario, USA	[29, 95]
Predatory fish	Tertiary consumer	Freshwater	Biomagnification factor	Up to 10	Lake Ontario, USA	[29, 95]
Aquatic invertebrates	Various	Freshwater	Biota‐sediment accumulation factor	0.5–3 (dimensionless)	Freshwater sediments	[95]
Aquatic plants (*Vallisneria natans*)	Primary producer	Freshwater	Tissue concentration	0.1–10 μg/kg	China	[90]
Crustaceans *(Procambarus clarkii)*	Secondary consumer	Freshwater	Tissue concentration	100–200 μg/kg	China	[91]
Terrestrial insects *(Drosophila melanogaster)*	Consumer	Terrestrial	Developmental toxicity (bioaccumulation implied)	Not quantified	Laboratory studies	[16]

#### 8.3.2. Carcinogenic and Genotoxic Risks to Humans From Environmental NDMA Exposure

**Figure 1:**
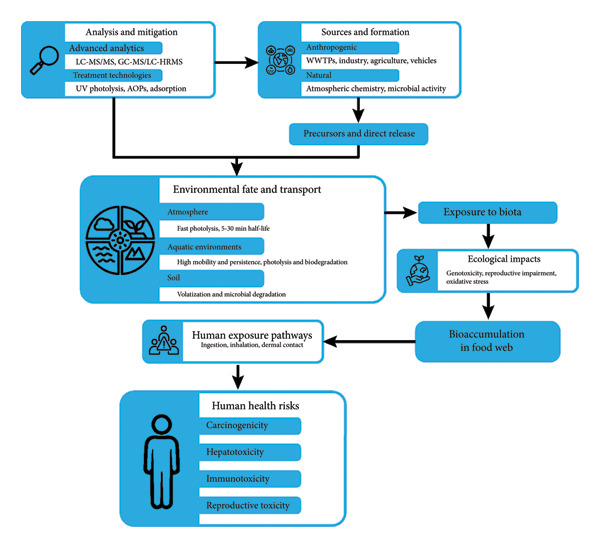
Environmental pathways, human exposure routes, and analytical detection of NDMA. The illustration depicts the primary anthropogenic sources (industrial emissions, WWTPs: wastewater treatment plants, and pharmaceutical production), environmental transport through air, water, and soil, bioaccumulation in aquatic and terrestrial food webs, and major human exposure routes. Mitigation by UV: ultraviolet photocatalysis, adsorption, and AOPs: advanced oxidation processes. Key analytical techniques (GC‐MS: gas chromatography‐mass spectrometry, LC‐MS/MS: liquid chromatography‐mass spectrometry/mass spectrometry, and LC‐HRMS: liquid chromatography‐high resolution mass spectrometry) used for NDMA detection and monitoring.

NDMA’s carcinogenic and genotoxic properties have been widely documented. Its primary mechanism of toxicity involves the induction of DNA adducts, disrupting key regulatory genes involved in cellular proliferation. As a result, NDMA has been classified as a probable human carcinogen (Group 2A) by the IARC, based on strong associations with cancers of the liver, lungs, and kidneys [96, 97]. Chronic exposure also induces oxidative stress, exacerbating DNA damage and promoting malignant transformation. Beyond oncogenic outcomes, NDMA exposure is implicated in immunotoxicity, reproductive dysfunction, and potential neurotoxicity. Vulnerable populations, including occupationally exposed individuals, children, pregnant women, and immunocompromised persons, are particularly at risk [16]. These findings highlight the imperative for robust and comprehensive regulatory strategies to mitigate exposure and safeguard public health. Figure 1 is used to support the engagement of readers and to achieve a greater clarity of the concepts by giving a combined visualized summary of the environmental behavior and detection pathways of NDMA.

The illustration charts the most significant anthropogenic contributors to NDMA, such as the industrial processes and wastewater treatment, and tracks the flow of the contaminant through major environmental compartments, such as water, soil, and biota. In this flowchart, source regions, environmental transportation, and patterns of occurrence are visually connected with the analytical tools necessary to detect them. This offers the readers an opportunity to understand the complexity of the NDMA fate by displaying all these linked processes and by showing how environmental occurrence, pathways to exposure, and the tools to analyze the data are functionally interdependent.

## 9. Conclusion

NDMA is a ubiquitous and dangerous environmental pollutant that has high mobility, persistence, and a strong genotoxic potential. Although much has been done in terms of comprehending its sources, the environmental behavior, and health effects, there still exist gaps in crucial knowledge. In order to be successful in suppressing the NDMA‐related hazards, future work should go beyond the retrospective examination and focus on the proactive, integrative approaches. The following are some of the major research and action priorities that are necessary to enhance global risk management and regulations.

Complex predictive and monitoring devices: Build and test in silico models (QSAR, molecular docking, and machine learning) to predict the potential to form NDMA, ecological toxicity, and risks to human health in a variety of environmental and product matrices. It requires harmonized, high‐throughput analytic techniques and real‐time sensors to be able to monitor widely at low cost, particularly in low‐resource environments.

Transgenerational and ecotoxicological research: Increase ecotoxicological studies in underrepresented terrestrial ecosystems and chronic exposure at low doses. Explore transgenerational impacts, interactions with cocontaminating chemicals, effects on soil microbial communities, and ecosystem processes.

Green engineering and source control: Focus on minimizing the NDMA precursors at the origin using the green chemistry principles in industry, pharmaceutical, and agriculture. Calculate and commercialize treatment technologies that are economically efficient, including catalytic advanced oxidation, UV‐based, and selective adsorption, to be deployed in a variety of water and wastewater systems.

Combined exposure and risk analysis: Standardize bioaccumulation measures and develop cumulative risk measurements, which consider multiple exposure pathways (water, food, and air) and populations at risk. Enhance epidemiological research to help demystify dose–response relationships on noncancer endpoints such as reproductive toxicity, immunological toxicity, and developmental toxicity.

Harmonization of regulating globally and capacity building: Promote global cooperation in order to harmonize regulatory boundaries, surveillance measures, and data reporting guidelines. Capacity building to support regions with weak monitoring facilities and the facilitation of open data exchange to inform evidence‐based policy and health guidance for the population.

The coordination of interdisciplinary and international actions to address these priorities will allow taking a more active and proactive approach to NDMA contamination, which will eventually guarantee the integrity of the ecological system and human health in a more polluted world.

## Funding

No funding was received for this manuscript.

## Conflicts of Interest

The authors declare no conflicts of interest.

## References

[1] Mustapha S. , Tijani J. O. , Ndamitso M. M. et al., The Occurrence of N-Nitrosodimethylamine (NDMA) in Swimming Pools: An Overview, Environmental Health Insights. (2021) 15, 10.1177/11786302211036520. PMC8335839 34376989

[2] Nawrocki J. and Andrzejewski P. , Nitrosamines and Water, The Journal of Hazardous Materials. (2011) 189, no. 1-2, 1–18, 10.1016/j.jhazmat.2011.02.005, 2-s2.0-79953685158. 21353742

[3] Chain E. P. o.C. i.t.F. , Schrenk D. , Bignami M. et al., Risk Assessment of N‐Nitrosamines in Food, EFSA Journal. (2023) 21, no. 3, 10.2903/j.efsa.2023.7884. PMC10043641 36999063

[4] Gold S. A. and Margulis V. , Carcinogenic Effects of Nitrosodimethylamine (NDMA) Contamination in Ranitidine: Defining the Relationship With Renal Malignancies, JU Open Plus. (2023) 1, no. 10, 10.1097/ju9.0000000000000058.

[5] Parr M. K. and Joseph J. F. , NDMA Impurity in Valsartan and Other Pharmaceutical Products: Analytical Methods for the Determination of N-Nitrosamines, Journal of Pharmacy Biomedicine Analytical. (2019) 164, 536–549, 10.1016/j.jpba.2018.11.010, 2-s2.0-85056644545. 30458387

[6] Aldawsari F. S. , Alshehry Y. M. , and Alghamdi T. S. , N-Nitrosodimethylamine (NDMA) Contamination of Ranitidine Products: A Review of Recent Findings, Journal of Food and Drug Analysis. (2021) 29, no. 1, 39–45, 10.38212/2224-6614.1133. 35696227 PMC9261846

[7] Eisenbrand G. , Buettner A. , Diel P. et al., Commentary of the SKLM to the EFSA Opinion on Risk Assessment of N-Nitrosamines in Food, Archives of Toxicology. (2024) 98, no. 6, 1573–1580, 10.1007/s00204-024-03726-1. 38573336 PMC11106120

[8] Garland K. , Adsorption Kinetics of N-Nitrosodimethylamine (NDMA) Onto Activated Carbons, 2020, New Mexico State University.

[9] Choi N. R. , Ahn Y. G. , Lee J. Y. et al., Particulate Nitrosamines and Nitramines in Seoul and Their Major Sources: Primary Emission Versus Secondary Formation, Environmental Science and Technology. (2021) 55, no. 12, 7841–7849, 10.1021/acs.est.1c01503. 34041906

[10] Li J. , Arnold W. A. , and Hozalski R. M. , Animal Feedlots and Domestic Wastewater Discharges are Likely Sources of N-Nitrosodimethylamine (NDMA) Precursors in Midwestern Watersheds, Environmental Science and Technology. (2024) 58, no. 6, 2973–2983, 10.1021/acs.est.3c09251. 38290429

[11] Meher A. K. and Zarouri A. , Environmental Applications of Mass Spectrometry for Emerging Contaminants, Molecules. (2025) 30, no. 2, 10.3390/molecules30020364. PMC11767766 39860232

[12] Chen W. , Li S. , Huang H. et al., Occurrence and Transport of N-Nitrosamines in the Urban Water Systems of the Pearl River Delta, Southern China, Science of the Total Environment. (2023) 890, 10.1016/j.scitotenv.2023.164251. 37201810

[13] Liu D. , Qin L. , Zeng H. et al., Ecotoxicological Risk Assessment of N-Nitrosamines to Selenastrum Capricornutum in Surface Waters: Insights Into Toxicity Mechanisms and Environmental Implications, Ecotoxicology and Environmental Safety. (2025) 296, 10.1016/j.ecoenv.2025.118179. 40253879

[14] Lu J.-X. , Lan H.-R. , Zeng D. et al., Design, Synthesis, Anticancer Activity and Molecular Docking of Quinoline-Based Dihydrazone Derivatives, RSC Advances. (2025) 15, no. 1, 231–243, 10.1039/d4ra06954d. 39758910 PMC11694625

[15] Sampaio G. M. , Nitrosamines Exposure and Metabolism: Toxicity Effects in Zebrafish, 2024.

[16] Tabares‐Mosquera O. E. , Juárez‐Díaz J. A. , Camacho‐Carranza R. , and Ramos‐Morales P. , Transgenerational Reproductive and Developmental Toxicity Induced by N‐Nitrosodimethylamine and Its Metabolite Formaldehyde in Drosophila melanogaster, Journal of Applied Toxicology. (2025) . 10.1002/jat.4749 PMC11982778 39775945

[17] Chowdhury S. , N-Nitrosodimethylamine (NDMA) in Food and Beverages: A Comparison in Context to Drinking Water, Human and Ecological Risk Assessment: An International Journal. (2014) 20, no. 5, 1291–1312, 10.1080/10807039.2013.817144, 2-s2.0-84897878354.

[18] Qiu Y. , Bei E. , Li X. et al., Quantitative Analysis of Source and Fate of N-Nitrosamines and Their Precursors in an Urban Water System in East China, The Journal of Hazardous Materials. (2021) 415, 10.1016/j.jhazmat.2021.125700. 34088188

[19] Song M. , Wang J. , DeNicola M. , and Hanigan D. , Natural Vs. Anthropogenic Sources of N-Nitrosodimethylamine Precursors in Surface Water, Water Research. (2024) 265, 10.1016/j.watres.2024.122313. 39197389

[20] Sgroi M. , Vagliasindi F. G. , Snyder S. A. , and Roccaro P. , N-Nitrosodimethylamine (NDMA) and Its Precursors in Water and Wastewater: A Review on Formation and Removal, Chemosphere. (2018) 191, 685–703, 10.1016/j.chemosphere.2017.10.089, 2-s2.0-85031999204. 29078192

[21] Chen Y. , Huang H. , Chen W. et al., Impact of Agricultural Activities on the Occurrence of N-Nitrosamines in an Aquatic Environment, Environmental Science: Processes & Impacts. (2024) 26, no. 3, 470–482, 10.1039/d3em00441d. 38282562

[22] Mitch W. A. and Sedlak D. L. , Characterization and Fate of N-Nitrosodimethylamine Precursors in Municipal Wastewater Treatment Plants, Environmental Science and Technology. (2004) 38, no. 5, 1445–1454, 10.1021/es035025n, 2-s2.0-1442300984. 15046346

[23] Environment Canada and Health Canada , Priority Substances List Assessment Report: N-Nitrosodimethylamine, 2001, Environment Canada and Health Canada.

[24] Krauss M. and Hollender J. , Analysis of Nitrosamines in Wastewater: Exploring the Trace Level Quantification Capabilities of a Hybrid Linear Ion Trap/Orbitrap Mass Spectrometer, Analytical Chemistry. (2008) 80, no. 3, 834–842, 10.1021/ac701804y, 2-s2.0-38849173797. 18183964

[25] López-Serna R. , Jurado A. , Vázquez-Suñé E. , Carrera J. , Petrović M. , and Barceló D. , Occurrence of 95 Pharmaceuticals and Transformation Products in Urban Groundwaters Underlying the Metropolis of Barcelona, Spain, Environmental Pollution. (2013) 174, 305–315, 10.1016/j.envpol.2012.11.022, 2-s2.0-84872143487. 23302545

[26] Spiegelhalder B. and Preussmann R. , Occupational Nitrosamine Exposure. 1. Rubber and Tyre Industry, Carcinogenesis. (1983) 4, no. 9, 1147–1152, 10.1093/carcin/4.9.1147, 2-s2.0-0020508681. 6883637

[27] Fajen J. M. , Carson G. A. , Rounbehler D. P. et al., *N*-Nitrosamines in the Rubber and Tire Industry, Science. (1979) 205, no. 4412, 1262–1264, 10.1126/science.472741, 2-s2.0-0018666645. 472741

[28] World Health Organization , N-Nitrosodimethylamine in Drinking-Water: Background Document for Development of WHO Guidelines for Drinking-Water Quality, 2008, World Health Organization.

[29] Chen Y. , Zeng H. , Huang H. et al., Occurrence and Fate of N-Nitrosamines in Full-Scale Domestic Wastewater Treatment Plants and Their Impact on Receiving Waters Along the Lijiang River, China, Journal of Hazardous Materials. (2024) 469, 10.1016/j.jhazmat.2024.133870. 38430594

[30] Harada K. , Microbialdegradation of Nitrosamines. II. Effect of the Conditions of Growth and Enzymatic Reaction on the Nitrosamine Breakdown, Bulletin of the Japanese Society of Scientific Flsheries. (1980) 6, 723–726.

[31] Zhou R. , Zhu K. , Gao Z. , Feng X. , Hu Q. , and Zhu L. , Formation Mechanisms of Carcinogenic N-Nitrosamines From Dissolved Organic Matter Derived From Nitrogen-Containing Microplastics During Chloramine Disinfection, Water Research. (2025) 281, 10.1016/j.watres.2025.123696. 40280007

[32] Zhao J. , Qi B. , Zhang P. et al., Research Progress on the Generation of NDMA by Typical PPCPs in Disinfection Treatment of Water Environment in China: A Review, Science of the Total Environment. (2024) 929, 10.1016/j.scitotenv.2024.172498. 38657805

[33] Shen L. , Liao X. , Gong Y. , Yan P. , Chen Z. , and Snyder S. A. , How to Control N-Nitrosodimethylamine (NDMA) in Water Treatment: From Origins to Removal, ACS ES&T Water. (2025) 5, no. 4, 1514–1529, 10.1021/acsestwater.4c01012.

[34] Moore G. , Personal Communication. Regulatory Affairs and Innovations Division, Pest Management Regulatory Agency, 1999, Health Canada.

[35] Li L. , Lin Q. , Liao X. et al., Novel Method for Reducing NDMA Formation From Daminozide During Ozonation: Performances and Mechanisms, Journal of Cleaner Production. (2024) 474, 10.1016/j.jclepro.2024.143485.

[36] Yang X. , Luo S. , Zhou J. et al., Ball-Milled Dysprosium Oxide Loaded Biochar-Montmorillonite Composite for Efficient Removal and Great Recycling Performance of Cationic Organic Pollutants, Industrial Crops and Products. (2025) 235, 10.1016/j.indcrop.2025.121777.

[37] Farré M. J. , Insa S. , Gernjak W. , Corominas L. , Čelić M. , and Acuña V. , N-Nitrosamines and Their Precursors in Wastewater Effluents From Selected Industries in Spain, The Journal of Hazardous Materials. (2023) 451, 10.1016/j.jhazmat.2023.131159. 36905908

[38] Bian Y. , Wang C. , Zhu G. , Ren B. , Zhang P. , and Hursthouse A. S. , Occurrence and Control of N-Nitrosodimethylamine in Water Engineering Systems, Environmental Engineering Research. (2019) 24, 1–16, 10.4491/eer.2018.021, 2-s2.0-85065191160.

[39] Alsayadi Y. M. , Dogra R. , Arora V. , and Shiven A. , Innovations in the Detection of N-Nitrosamine Impurities in Pharmaceuticals: Analytical and Regulatory Challenges, Critical Reviews in Analytical Chemistry. (2025) 1–26, 10.1080/10408347.2025.2512443. 40492544

[40] Manchuri K. M. , Shaik M. A. , Gopireddy V. S. R. , Sultana N. , and Gogineni S. , Analytical Methodologies to Detect N-Nitrosamine Impurities in Active Pharmaceutical Ingredients, Drug Products and Other Matrices, Chemical Research in Toxicology. (2024) 37, no. 9, 1456–1483, 10.1021/acs.chemrestox.4c00234. 39158368 PMC12135945

[41] Khan S. , Naushad M. , Govarthanan M. , Iqbal J. , and Alfadul S. M. , Emerging Contaminants of High Concern for the Environment: Current Trends and Future Research, Environmental Research. (2022) 207, 10.1016/j.envres.2021.112609. 34968428

[42] McMahon N. F. , Brooker P. G. , Pavey T. G. , and Leveritt M. D. , Nitrate, Nitrite and Nitrosamines in the Global Food Supply, Critical Reviews in Food Science and Nutrition. (2024) 64, no. 9, 2673–2694, 10.1080/10408398.2022.2124949. 36168920

[43] De Vocht F. , Burstyn I. , Straif K. et al., Occupational Exposure to NDMA and NMOR in the European Rubber Industry, Journal of Environmental Monitoring. (2007) 9, no. 3, 253–259, 10.1039/b615472g, 2-s2.0-33847736287. 17344951

[44] EPA U. , Technical Fact Sheet–N-Nitroso-Dimethylamine (NDMA), 2017, https://www.epa.gov/sites/default/files/2017-10/documents/ndma_fact_sheet_update_9-15-17_508.pdf.

[45] Sen N. P. , Formation and Occurrence of Nitrosamines in Food, Diet, Nutrition and Cancer: A Critical Evaluation. (2018) 135–160, 10.1201/9781351071383-9.

[46] Mitch W. A. , Sharp J. O. , Trussell R. R. , Valentine R. L. , Alvarez-Cohen L. , and Sedlak D. L. , N-Nitrosodimethylamine (NDMA) as a Drinking Water Contaminant: A Review, Environmental Engineering Science. (2003) 20, no. 5, 389–404, 10.1089/109287503768335896, 2-s2.0-0141717009.

[47] Schmidt C. K. and Brauch H.-J. N. , N-Dimethylsulfamide as Precursor for N-Nitrosodimethylamine (NDMA) Formation Upon Ozonation and Its Fate During Drinking Water Treatment, Environmental Science and Technology. (2008) 42, no. 17, 6340–6346, 10.1021/es7030467, 2-s2.0-50849109477. 18800499

[48] Zhang S. , Zhou Y. , Liu Y. D. , and Zhong R. , Reinvestigation of Ndma Formation Mechanisms From Tertiary Amines During Chloramination: A DFT Study, Environmental Sciences: Water Research & Technology. (2020) 6, no. 8, 2078–2088, 10.1039/d0ew00098a.

[49] Bie P. , Ji L. , Cui H. et al., A Review and Evaluation of Nonroad Diesel Mobile Machinery Emission Control in China, Journal of Environmental Sciences. (2023) 123, 30–40, 10.1016/j.jes.2021.12.041. 36521993

[50] EPA , Nitrosamines in Pesticides: Occurrence and Environmental Fate, 2021, USEPA.

[51] Kah M. , Weniger A.-K. , and Hofmann T. , Impacts of (Nano)Formulations on the Fate of an Insecticide in Soil and Consequences for Environmental Exposure Assessment, Environmental Science and Technology. (2016) 50, no. 20, 10960–10967, 10.1021/acs.est.6b02477, 2-s2.0-84991776554. 27648740 PMC5072106

[52] Ma Q. , Qian Y. , Su W. et al., Degradation of Agricultural Polyethylene Film by Greater Wax Moth (Galleria Mellonella) Larvae and Screening of Involved Gut Bacteria, Ecotoxicology and Environmental Safety. (2025) 303, 10.1016/j.ecoenv.2025.118841. 40829281

[53] Venkatesan A. K. , Pycke B. F. , and Halden R. U. , Detection and Occurrence of N-Nitrosamines in Archived Biosolids From the Targeted National Sewage Sludge Survey of the US Environmental Protection Agency, Environmental Science and Technology. (2014) 48, no. 9, 5085–5092, 10.1021/es5001352, 2-s2.0-84899849525. 24697330 PMC4018098

[54] Zha X. , Wang S. , and Zhang D. , Reductive Degradation of N-Nitrosodimethylamine Via UV/Sulfite Advanced Reduction Process: Efficiency, Influencing Factors and Mechanism, Water. (2023) 15, no. 20, 10.3390/w15203670.

[55] Fournier D. , Hawari J. , Streger S. H. , McClay K. , and Hatzinger P. B. , Biotransformation of N-Nitrosodimethylamine by Pseudomonas Mendocina KR1, Applied and Environmental Microbiology. (2006) 72, no. 10, 6693–6698, 10.1128/aem.01535-06, 2-s2.0-33750068601. 16950909 PMC1610310

[56] Leavey-Roback S. L. , Krasner S. W. , and Suffet I. H. M. , The Effect of Natural Organic Matter Polarity and Molecular Weight on NDMA Formation From Two Antibiotics Containing Dimethylamine Functional Groups, Science of the Total Environment. (2016) 572, 1231–1237, 10.1016/j.scitotenv.2016.08.041, 2-s2.0-84981719019. 27522283

[57] Zhang J. , Investigations Into the Occurrence, Formation and Fate of N-Nitrosodimethylamine (NDMA) in Air and Water, 2016, Arizona State University.

[58] Plumlee M. H. and Reinhard M. , Photochemical Attenuation of N-Nitrosodimethylamine (NDMA) and Other Nitrosamines in Surface Water, Environmental Science and Technology. (2007) 41, no. 17, 6170–6176, 10.1021/es070818l, 2-s2.0-34548580385. 17937298

[59] Wen Y. , Zhang S. , Wu Y. , and Hao J. , Vehicular Ammonia Emissions: An Underappreciated Emission Source in Densely Populated Areas, Atmospheric Chemistry and Physics. (2023) 23, no. 6, 3819–3828, 10.5194/acp-23-3819-2023.

[60] ATSDR , Toxicological Profile for N-Nitrosodimethylamine, 2023, U.S. Department of Health and Human Services, ATSDR.

[61] Reny R. , Plumlee M. H. , Kodamatani H. , Suffet I. H. M. , and Roback S. L. , NDMA and NDMA Precursor Attenuation in Environmental Buffers Prior to Groundwater Recharge for Potable Reuse, Science of the Total Environment. (2021) 762, 10.1016/j.scitotenv.2020.144287. 33360455

[62] Patterson B. M. , Pitoi M. M. , Furness A. J. , Bastow T. P. , and McKinley A. J. , Fate of N-Nitrosodimethylamine in Recycled Water After Recharge Into Anaerobic Aquifer, Water Research. (2012) 46, no. 4, 1260–1272, 10.1016/j.watres.2011.12.032, 2-s2.0-84856112521. 22244272

[63] Arienzo M. , Gan J. , Ernst F. , Qin S. , Bondarenko S. , and Sedlak D. , Loss Pathways of N‐Nitrosodimethylamine (NDMA) in Turfgrass Soils, Journal of Environmental Quality. (2006) 35, no. 1, 285–292, 10.2134/jeq2005.0265, 2-s2.0-31844455596. 16397104

[64] Yang W. , Gan J. , Liu W. , and Green R. , Degradation of N‐Nitrosodimethylamine (NDMA) in Landscape Soils, Journal of Environmental Quality. (2005) 34, no. 1, 336–341, 10.2134/jeq2005.0336. 15647563

[65] Zhang Q. , Zhang G. , Yin P. et al., Toxicological Effects of Soil Contaminated With Spirotetramat to the Earthworm Eisenia Fetida, Chemosphere. (2015) 139, 138–145, 10.1016/j.chemosphere.2015.05.091, 2-s2.0-84942522278. 26081578

[66] Szczuka A. , Huang N. , MacDonald J. A. , Nayak A. , Zhang Z. , and Mitch W. A. , N-Nitrosodimethylamine Formation During UV/Hydrogen Peroxide and UV/Chlorine Advanced Oxidation Process Treatment Following Reverse Osmosis for Potable Reuse, Environmental Science and Technology. (2020) 54, no. 23, 15465–15475, 10.1021/acs.est.0c05704. 33185421

[67] OEHHA , Draft Public Health Goal for N-Nitrosodimethylamine (NDMA) in Drinking Water, 2025, OEHHA, California Environmental Protection Agency.

[68] WHO , Guidelines for Drinking-Water Quality, 2011.

[69] Cioc R. C. , Joyce C. , Mayr M. , and Bream R. N. , Formation of N-Nitrosamine Drug Substance Related Impurities in Medicines: A Regulatory Perspective on Risk Factors and Mitigation Strategies, Organic Process Research & Development. (2023) 27, no. 10, 1736–1750, 10.1021/acs.oprd.3c00153.

[70] Topuz E. , Aydin E. , and Pehlivanoglu-Mantas E. , A Practical LC-MS/MS Method for the Detection of NDMA at Nanogram Per Liter Concentrations in Multiple Water Matrices, Water, Air, & Soil Pollution. (2012) 223, no. 9, 5793–5802, 10.1007/s11270-012-1315-1, 2-s2.0-84870252874.

[71] Agency E. M. , Nitrosamine Impurities, https://www.ema.europa.eu/en/human-regulatory-overview/post-authorisation/pharmacovigilance-post-authorisation/referral-procedures-human-medicines/nitrosamine-impurities.

[72] Qamar S. , Ahmad M. , Hussain K. et al., Risk Assessment of Amine-Based Anti-Hypertensive Drugs for the Possible Presence of N-Nitroso Dimethyl Amine by a Verified GC-MS Method, Pakistan Journal of Pharmaceutical Sciences. (2025) 38, no. 2, 559–569. 40501254

[73] Shomali F. , Talebpour Z. , Abedi G. et al., Analysis and Risk Assessment of Nitrosamines in Sartans Using GC-MS and Monte Carlo Simulation, Scientific Reports. (2025) 15, no. 1, 10.1038/s41598-025-97844-0. PMC12144120 40481262

[74] Ali H. M. , Alsohaimi I. H. , Rizwan Khan M. et al., Selective and Sensitive GC-MS Analysis of Carcinogenic N-Nitrosodimethylamine in Pharmaceuticals Using a Magnetic Coconut Carbon Composite as a Solid-Phase Extraction Sorbent, Journal of Taibah University for Science. (2023) 17, no. 1, 10.1080/16583655.2023.2254352.

[75] Tummala S. R. and Amgoth K. P. , Development of GC-MS/MS Method for Simultaneous Estimation of Four Nitrosoamine Genotoxic Impurities in Valsartan, Turkish Journal of Pharmaceutical Sciences. (2022) 19, no. 4, 455–461, 10.4274/tjps.galenos.2021.17702. 36047600 PMC9438752

[76] Sungur Ş. , Aljoubasi M. , and Aydın Z. , Determination of N-Nitrosodimethyl Amine (NDMA) and N-Nitrosodiethyl Amine (NDEA) in Medicines Containing Sartan and Its Derivatives, Journal of Faculty of Pharmacy of Ankara University. (2024) 48, no. 3, 1118–1127, 10.33483/jfpau.1436182.

[77] King F. J. , Searle A. D. , and Urquhart M. W. , Ranitidine—Investigations Into the Root Cause for the Presence of N-Nitroso-N,N-dimethylamine in Ranitidine Hydrochloride Drug Substances and Associated Drug Products, Organic Process Research & Development. (2020) 24, no. 12, 2915–2926, 10.1021/acs.oprd.0c00462.

[78] De Palma R. , Patel V. , Florian J. et al., A Bioanalytical Method for Quantification of N-Nitrosodimethylamine (NDMA) in Human Plasma and Urine With Different Meals and Following Administration of Ranitidine, Journal of Pharmaceutical Sciences. (2023) 112, no. 5, 1315–1323, 10.1016/j.xphs.2023.01.026. 36736776

[79] Li N. , Yuan M. , and Che J. , Development and Validation of UPLC-MS/MS Method for Icariin and Its Metabolites in Mouse Urine, Frontiers in Pharmacology. (2024) 15, 10.3389/fphar.2024.1389754. PMC11196403 38919252

[80] Administration USFAD , Liquid Chromatography-High Resolution Mass Spectrometry (LC-HRMS) Method for the Determination of Six Nitrosamine Impurities in Arb Drugs, 2019, U.S. Food and Drug Administration (FDA).

[81] Li X.-Y. , Wu W.-F. , Wu C.-Y. et al., Seeds Act as Vectors for Antibiotic Resistance Gene Dissemination in a Soil-Plant Continuum, Environmental Science and Technology. (2023) 57, no. 50, 21358–21369, 10.1021/acs.est.3c05678. 38078407

[82] Yamamoto E. , Kan-No H. , Tomita N. , Ando D. , Miyazaki T. , and Izutsu K.-I. , Isolation of N-Nitrosodimethylamine From Drug Substances Using Solid-Phase extraction-Liquid Chromatography–Tandem Mass Spectrometry, Journal of Pharmacy Biomedicine Analytical. (2022) 210, 10.1016/j.jpba.2021.114561. 34974238

[83] Tarawneh I. N. , Shmeis R. A. , Alfuqaha S. M. , and Al Omari M. M. , Determination of N-Nitrosodimethyl Amine Impurity in Valsartan by HPLC and LC-MS/MS Methods, Chinese Journal of Analytical Chemistry. (2022) 50, no. 11, 10.1016/j.cjac.2022.100150.

[84] Kaly M. K. , Rahman M. E. , Rana M. S. , Acharjee U. K. , and Nasirujjaman K. , Genotoxic Effects of NDMA-Contaminated Ranitidine on Allium Cepa Cells and Unveiling Carcinogenic Mechanisms Via DFT and Molecular Dynamics Simulation Study, Scientific Reports. (2024) 14, no. 1, 10.1038/s41598-024-82984-6. PMC11682305 39733169

[85] Daniş İ. , Agar S. , Yurtsever M. , and Ünal D. Ö. , High Performance Liquid Chromatography-Tandem Mass Spectrometric Determination of Carcinogen Nitrosamine Impurities From Pharmaceuticals and DNA Binding Confirmation Aided by Molecular Docking Application, İstanbul Journal of Pharmacy. (2024) 54, no. 3, 386–394, 10.26650/istanbuljpharm.2024.1500047.

[86] Isiyaka H. , Jumbri K. , Sambudi N. , Zango Z. , Abdullah N. , and Saad B. , Optimizations and Docking Simulation Study for Metolachlor Adsorption From Water Onto Mil-101 (Cr) Metal–Organic Framework, International Journal of Environmental Science and Technology. (2023) 20, no. 1, 277–292, 10.1007/s13762-022-04059-1.

[87] Fahrer J. and Christmann M. , DNA Alkylation Damage by Nitrosamines and Relevant DNA Repair Pathways, International Journal of Molecular Sciences. (2023) 24, no. 5, 10.3390/ijms24054684. PMC10003415 36902118

[88] Reh B. D. , DeBord D. G. , Butler M. A. , Reid T. M. , Mueller C. , and Fajen J. M. , O 6-Methylguanine DNA Adducts Associated With Occupational Nitrosamine Exposure, Carcinogenesis. (2000) 21, no. 1, 29–33, 10.1093/carcin/21.1.29. 10607730

[89] Jiang W. , Liang Y. , Han M. et al., RNF144A and RNF144B: Important Molecules for Health, Open Life Sciences. (2025) 20, no. 1, 10.1515/biol-2025-1130. PMC12317654 40756570

[90] Huang K. , Huang H. , Huang X. , Lao A. , Zheng Z. , and Wu H. , Combined Toxic Effects and Mechanisms of Chloroacetic Acid and N-Nitrosodimethylamine on Submerged Macrophytes, Water. (2024) 16, no. 18, 10.3390/w16182689.

[91] Brooks S. , The Toxicity of Selected Amines and Secondary Products to Aquatic Organisms: A Review NIVA Report 5698-2008, 2008, Norwegian Institute for Water Research (NIVA).

[92] Lan T. , Hu Y. , Cheng L. et al., Floods and Diarrheal Morbidity: Evidence on the Relationship, Effect Modifiers, and Attributable Risk From Sichuan Province, China, Journal of Global Health. (2022) 12, 10.7189/jogh.12.11007. PMC9308977 35871400

[93] Gushgari A. J. and Halden R. U. , Critical Review of Major Sources of Human Exposure to N-Nitrosamines, Chemosphere. (2018) 210, 1124–1136, 10.1016/j.chemosphere.2018.07.098, 2-s2.0-85053175824. 30208538

[94] Gushgari A. J. , Halden R. U. , and Venkatesan A. K. , Occurrence of N-Nitrosamines in US Freshwater Sediments Near Wastewater Treatment Plants, The Journal of Hazardous Materials. (2017) 323, 109–115, 10.1016/j.jhazmat.2016.03.091, 2-s2.0-84975514772. 27067539

[95] Arnot J. A. and Gobas F. A. , A Food Web Bioaccumulation Model for Organic Chemicals in Aquatic Ecosystems, Environmental Toxicology and Chemistry. (2004) 23, no. 10, 2343–2355, 10.1897/03-438, 2-s2.0-4644229906. 15511097

[96] Smoke T. and Smoking I. , IARC Monographs on the Evaluation of Carcinogenic Risks to Humans, IARC. (2004) 1, 1–1452. PMC4781536 15285078

[97] Zhao B. , Wong Y. , Ihara M. et al., Characterization of Nitrosamines and Nitrosamine Precursors as Non-Point Source Pollutants During Heavy Rainfall Events in an Urban Water Environment, The Journal of Hazardous Materials. (2022) 424, 10.1016/j.jhazmat.2021.127552. 34736194

